# Spontaneous and Perturbational Complexity in Cortical Cultures

**DOI:** 10.3390/brainsci11111453

**Published:** 2021-11-01

**Authors:** Ilaria Colombi, Thierry Nieus, Marcello Massimini, Michela Chiappalone

**Affiliations:** 1 Brain Development and Disease Laboratory, Istituto Italiano di Tecnologia, 16163 Genova, Italy; ilaria.colombi@iit.it; 2 Department of Biomedical and Clinical Sciences “L. Sacco”, University of Milan, 20157 Milan, Italy; thierry.nieus@unimi.it (T.N.); marcello.massimini@unimi.it (M.M.); 3 IRCCS, Fondazione Don Carlo Gnocchi, 20148 Milan, Italy; 4 Department of Informatics, Bioengineering, Robotics and System Engineering, 16145 Genova, Italy; 5 Rehab Technologies Lab., Istituto Italiano di Tecnologia, 16163 Genova, Italy

**Keywords:** in vitro, micro-electrode array (MEA), cortical networks, complexity, perturbational complexity index (PCI), spikes, local field potentials (LFP), electrical stimulation

## Abstract

Dissociated cortical neurons in vitro display spontaneously synchronized, low-frequency firing patterns, which can resemble the slow wave oscillations characterizing sleep in vivo. Experiments in humans, rodents, and cortical slices have shown that awakening or the administration of activating neuromodulators decrease slow waves, while increasing the spatio-temporal complexity of responses to perturbations. In this study, we attempted to replicate those findings using in vitro cortical cultures coupled with micro-electrode arrays and chemically treated with carbachol (CCh), to modulate sleep-like activity and suppress slow oscillations. We adapted metrics such as neural complexity (NC) and the perturbational complexity index (PCI), typically employed in animal and human brain studies, to quantify complexity in simplified, unstructured networks, both during resting state and in response to electrical stimulation. After CCh administration, we found a decrease in the amplitude of the initial response and a marked enhancement of the complexity during spontaneous activity. Crucially, unlike in cortical slices and intact brains, PCI in cortical cultures displayed only a moderate increase. This dissociation suggests that PCI, a measure of the complexity of causal interactions, requires more than activating neuromodulation and that additional factors, such as an appropriate circuit architecture, may be necessary. Exploring more structured in vitro networks, characterized by the presence of strong lateral connections, recurrent excitation, and feedback loops, may thus help to identify the features that are more relevant to support causal complexity.

## 1. Introduction

The ability of the brain (and the nervous system in general) to produce different actions in response to several sensory stimuli depends, above and beyond single-cell specialization, on the way neurons are connected with each other in local circuits and long-range networks [1]. To this end, the study of the properties and mechanisms of neuronal interactions, both in vivo and in vitro, is fundamental for the understanding of the brain’s function. In this context, neurosciences have evolved in recent decades, facing the complexity of the brain and of the nervous system with a multi-level approach, involving not only many different branches of biology (molecular and cellular biology, genetics, biochemistry, etc.), but also physics, mathematics, engineering, and related fields. Studying brain complexity and advancing our understanding of its function involves both innovations in experimental methods (to observe and perturb brain activity) and in computational tools (to analyze recorded data). Among the different electrophysiological techniques, micro-electrode array (MEA) technology represents a powerful tool that enables the long-term recordings of local field potentials (LFPs) and extracellular action potentials (i.e., spikes) from a population of neurons. Multisite extracellular recordings of neuronal cell cultures coupled with MEA have already demonstrated their potential to reveal a wide range of neural phenomena, from individual cell behaviors [2,3] to network dynamics [4], either in physiological [5,6] or pathological conditions [7]. In addition, like in vivo networks, cell cultures are capable of processing and segregating spatial–temporal input stimuli as a function of the network state [8]. Moreover, an extended information theory framework allowed the quantification of the extent of state dependency in cell culture responses recorded with high-density MEAs [9]. The latter findings [8,9] well connect to theoretical works about reservoir computing and echo-state networks [10], showing that cell cultures represent an ideal, yet simple, system to investigate neural activity at the mesoscale network level [11,12].

Integration and segregation are key organizing principles of brain architecture and an optimal balance between the two within cortical networks is considered as a fundamental prerequisite for consciousness [13,14,15]. The arrangement of local and large-scale connectivity across neuronal elements is a key determinant for optimizing the joint presence of segregation and integration and for attaining high levels of brain complexity [14,15,16,17]. Specifically, strong local links, patchiness in the connectivity, and large numbers of short reentrant circuits together with specific long-range connections have been identified as fundamental prerequisites [15,18]. However, such structural properties need to be immersed in the appropriate milieu of activating neuromodulators in order to express high levels of complexity. Accordingly, perturbations with transcranial magnetic stimulation (TMS) evoke a chain of recurrent, complex activations in the cortex, as measured by electroencephalography (EEG), in wakefulness but not during NREM sleep [19]. In subsequent studies the complexity of brain responses to direct cortical stimuli, quantified by the perturbational complexity index (PCI) [20] was tested across many different conditions in humans [20,21,22], rodents [23,24], and cortical slices [25,26]. While these experiments provided fundamental insights on the effects of neuromodulation, anesthetics, and brain lesions on PCI, the role of basic cytoarchitectonics and local connectivity has never been explored.

In the present work, we provide a first exploration of these aspects by applying measures of complexity to in vitro networks of neurons cultured over MEAs during pharmacological manipulations mimicking the effects of activating neuromodulation, as in [27]. We administered carbachol (CCh) to the neuronal cultures, a cholinergic receptor agonist that depresses evoked excitatory postsynaptic potentials (EPSPs) and evokes inhibitory postsynaptic potentials (IPSPs), thus altering the excitatory–inhibitory balance [28,29]. We measured both spontaneous activity and responses to perturbations using approximate measures previously employed to assess complexity in human subjects during wakefulness, sleep, anesthesia, and disorders of consciousness, as well as in rodents and cortical slices during pharmacological manipulation [21]. Specifically, we aimed at exploring the effects of activating neuromodulation on dissociated cell cultures, a model of neuronal circuits that can show rich activity patterns but lacks the typical architecture of the cortex.

We found that CCh was effective in increasing the complexity of spontaneous activity with respect to basal conditions. Consistent with previous experiments in humans and animals, we also found that activating neuromodulation decreased the amplitude of the initial oscillatory response. However, during CCh administration, the overall spatio-temporal pattern of the spiking-evoked response remained similar to that observed in the baseline condition, resulting in a moderate increase in PCI. This result suggests that additional properties of the network (e.g., recurrent connectivity, feedback loops) are needed to better mimic the changes in the spatio-temporal patterns observed in human recordings, rodents, and cortical slices across brain states. Our findings thus prompt additional efforts to develop more structured in vitro systems able to better recapitulate the cellular and network mechanisms and determining the richness and complexity of activity patterns found in higher systems.

## 2. Materials and Methods

### 2.1. Cell Cultures

Cortical cell cultures were prepared from embryonic rats at gestational day 18 (pregnant Sprague Dawley female rats delivered by Charles River Laboratories, Lecco, Italy). All experimental procedures and animal care were conducted in conformity with institutional guidelines, in accordance with the European legislation (European Communities Directive of 24 November 1986, 86/609/EEC) and with the NIH Guide for the Care and Use of Laboratory Animals. The procedure for preparing the cultures is reported in previous studies [30,31]. Briefly, the cerebral cortices of 4–5 embryos were dissected out from the brain and dissociated by enzymatic digestion in 5 mL of trypsin 0.125% and HBSS-diluted DNAsi 0.25 mg/mL (Sigma-Aldrich, Saint Louis, MO, USA) at 37 °C for 30 min. Trypsin digestion was blocked using 5 mL of Neurobasal medium (Thermo Fisher Scientific, Waltham, MA, USA) containing 2% of B27 supplement, 1% penicillin/streptomycin, 1% L-glutamine (Thermo Fisher Scientific, Waltham, MA, USA), plus 10% of heat-inactivated FBS (Thermo Fisher Scientific, Waltham, MA, USA). Cells were centrifuged for 5 min at 1200 rpm and then resuspended by pipetting in 2–3 mL of complete Neurobasal medium plus FBS. Cell debris was removed by centrifuging at 700 rpm for 7 min. After resuspension in complete culture medium, neurons were counted with trypan blue dye (Sigma-Aldrich, Saint Louis, MO, USA) and then plated on 60-channel planar MEAs (Multi Channel Systems, MCS, Reutlingen, Germany) that had been previously coated with borate buffer and poly-L-lysine to promote cell adhesion (final cell density approximately 1200 cells/mm^2^). Cells were finally placed in a humidified incubator having an atmosphere of 5% CO_2_—95% air at 37 °C. Their maintenance was guaranteed by 1 mL of nutrient medium (i.e., serum-free Neurobasal medium supplemented with B27 and Glutamax-I), 50% of which was changed weekly.

**Figure 1:**
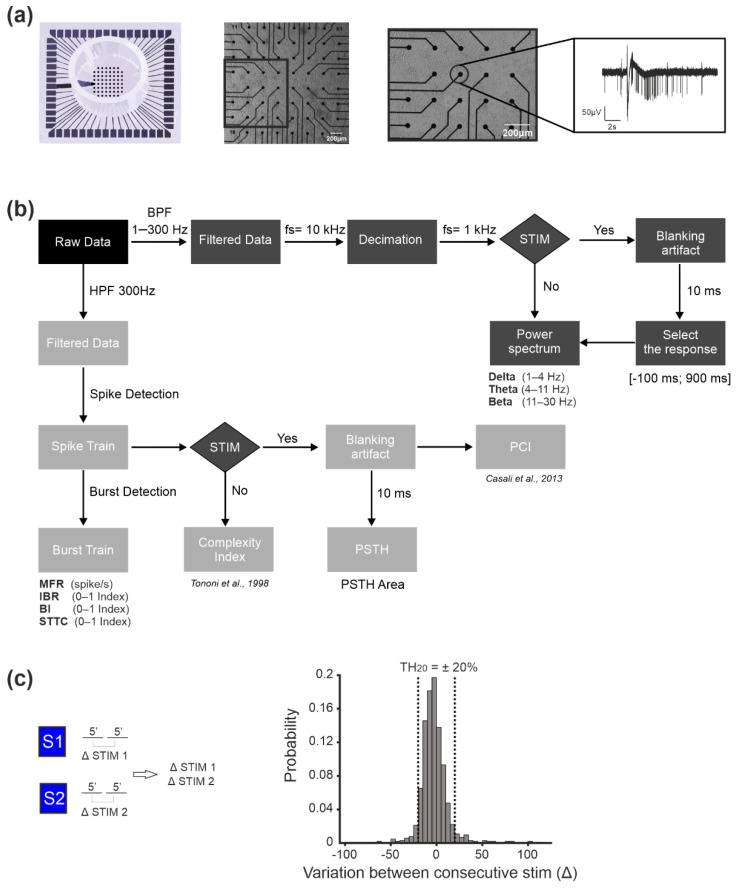
Methods of our experiments. (**a**) From left to right: cartoon of a 60–channel micro-electrode array (MEA) by MCS with a standard electrode layout (8 × 8); optical micrograph of a MEA plated with cortical cultures (scale bar 200 µm); zoom of the squared area highlighted in the previous picture (scale bar 200 µm); a typical raw trace recorded from a representative micro-electrode of the MEA. (**b**) Scheme of the data analysis workflow. Data were acquired by means of the MC_Rack software (MCS). We then processed it by following two approaches: (i) by band pass filtering in the frequency band 1–300 Hz, we obtained filtered data which was then decimated at 1 kHz, i.e., the local field potentials (LFPs); (ii) by high pass filtering (>300 Hz) we obtained filtered data which were processed by means of a spike detection algorithm, to obtain the multi-unit activity (MUA). The MUA was further analyzed to detect firing and bursting parameters and to compute the correlation index, in the form of the spike time tiling coefficient (STTC). For both (i) and (ii), we analyzed the spontaneous as well as the evoked activity (see Methods for details on the analysis). (**c**) Selection of the PSTH threshold during the stability phases (see Methods). On the left, the variation in the PSTH area in the basal condition is computed by considering the responses to stimulation in the first and in the second five-minute time frames. The variation in the percentage of the PSTH area for all the channels in all the experiments was then plotted as a histogram (rigth panel) which served to define the ‘natural’ fluctuation range of the considered parameter. The threshold was then defined as the mean ± 1SD of the PSTH area variation of the obtained histogram, which resulted I being equal to ± 20%. We then named that threshold TH_20_.

Figure 1a shows a representative MEA used for this study and a culture of neurons grown over its surface, together with the typical bursting pattern (cf. ‘Data Analysis’) exhibited by the recording channels.

### 2.2. Experimental Set-Up and Micro-Electrode Array Recordings

The MEAs used in this study (Multichannel Systems, MCS, Reutlingen, Germany) are characterized by planar microelectrodes arranged in an 8 × 8 layout, excluding corners and one reference electrode, for a total of 59 TiN/SiN round recording electrodes (30 µm diameter; 200 µm center-to-center inter electrode distance). Recordings were performed by means of the MEA60 System (MCS), composed by the Multichannel System amplifier (bandwidth 1 Hz–3 kHz) and the Multichannel System data acquisition card. The signal from each channel was sampled at 10 kHz. The online monitoring and recording of data was performed by the MC_Rack software (MCS). To reduce the thermal stress of the cells during the experiment, MEAs were kept at 37 °C by means of a controlled thermostat (MCS) and covered by custom-made PDMS caps to avoid evaporation and prevent changes in osmolarity.

### 2.3. Experimental Protocol

After a period of rest (~30 min) outside the incubator to allow the culture to adapt to the new environment and reach a stable level of activity, the experiment started. The protocol included 30 min of basal recording (i.e., in Neurobasal medium) followed by two sessions of electrical stimulation (10 min each) from two different sites. The sites were chosen independently for each culture and the two sites able to evoke the most prominent responses were selected, according to the same approach adopted in our previous studies [6,32]. We then applied the cholinergic agonist carbachol (CCh, 20 µM), as reported in previous studies [27,29], by directly pipetting it into the culture medium and recorded from the cultures for 1 h in the absence of electrical stimulation. Since we observed that mechanical perturbation caused by the addition of any substance to the medium through a pipette could cause temporary instability of the firing rate, we discarded the first 10 min of recording following CCh administration. After one hour, we repeated the electrical stimulation protocol in the presence of CCh, using the same locations for stimulation and performing the stimulation in the same order that was used in the basal recording. This allowed us to compare the evoked response at the baseline (i.e., in Neurobasal medium) and during the drug treatment, to evaluate whether and how the presence of CCh influenced the evoked response of the network. For each stimulation session (duration 10 min), a total of 120 trials per stimulation site were collected. The stimulus shape was the same across all experiments and all experimental phases (biphasic voltage pulse, duration 600 µs, 750 mV half-amplitude every 5 s). These parameters have been shown to be the most effective ones for evoking responses in neurons cultured on MEAs [33], without inducing any form of long-term change [34,35]. A total of 9 experiments were performed using mature cultures in the 5th–7th week in vitro.

### 2.4. Data Analysis

There are two main types of extracellular signals which can be recorded from a network of neurons: local field potentials (LFPs) and multi-unit activity (MUA). LFPs (<300 Hz) constitute the low-frequency component of the raw signal and are related to the activity of an entire neuronal population, responsible for the generation of neural oscillations in the recorded network [36]. The MUA constitutes the high-frequency portion of the raw signal (>300 Hz) and represents the spiking activity of the neurons nearby the recording electrodes [37]. In this study, we analyzed both LFP and MUA, by following the methodology reported in the scheme of Figure 1b and briefly described below.

#### 2.4.1. Local Field Potential (LFP) Analysis

To select the LFP components, we low-pass-filtered the raw data between 1 and 300 Hz. We then computed the power spectral density (PSD) of the decimated signal (sampling frequency 1 kHz) (μV^2^/Hz) using the Welch method (Windows = 5 s, overlap = 50%). We considered only the lower frequency bands of the signal, in particular the delta (1–4 Hz), theta (4–11 Hz), and beta (11–30 Hz) bands, as in our previous study [27]. To characterize the LFP, we calculated the power in each of those frequency bands for all the electrodes of the MEA in the dataset. Then, we averaged the power for all the electrodes for each recorded MEA to obtain a mean value for each frequency band.

For the stimulation phase, we computed the mean response to the stimulation pulse for the top 20 channels for each culture that displayed the highest MFR (mean firing rate, spikes/s). We then considered the response to the stimulation (100 ms before and 900 ms after the stimulus) and repeated the analysis described above, computing the power in each frequency band. To eliminate the artifact, we discarded the first 10 ms after the stimulus. PSD was computed using the Pwelch function in MATLAB with the following parameters: win = 200 ms, overlap = 50%, Fs = 10^3^ Hz and Max frequency = 100 Hz.

#### 2.4.2. Multi-Unit Activity (MUA) Analysis

In the high-pass filtered data (f > 300 Hz), we detected spikes (i.e., single over-threshold peaks) and bursts (i.e., groups of tightly packed spikes) using custom software developed in our or other laboratories [38,39,40,41]. We then computed the following electrophysiological parameters, which were also used in our previous work [27]: mean firing rate (MFR, spikes/s), inverse burst ratio (IBR, percentage of spikes outside the burst), and burstiness index (BI, index of the burstiness level of the network, as described in [41]). The BI index is normalized between ‘0’ (no bursts) and ‘1’ (burst-dominated network) values. We normalized each experiment with respect to the mean of the selected parameter (i.e., MFR, IBR, and BI) during the basal recording.

Since we observed that the electrophysiological parameters reached steady-state values after 30 min of CCh administration, we divided the CCh recording phase into two data-driven phases, namely CCh1 and CCh2 (i.e., the first 30 min and the last 20 min, respectively).

To characterize the amount of synchronization inside each neuronal network we used the spike time tiling coefficients (STTCs, [42]). We computed the STTCs using the function sttc of the Python module Elephant [43]. In order to speed up the computations of the STTC, we optimized the existing code, achieving a speed factor of ~200 on a DELL XPS laptop equipped with an Intel^®^ Core™ i7 Processor. The modified code is available on Github (https://github.com/thierrynieus/Spike-Time-Tiling-Coefficient, accessed date: 29 October 2021). Then, we also verified that the result was consistent with another measure used in the literature to quantify spike synchrony. We quantified synchrony with the parameter-free SPIKE synchronization measure [44] implemented in the python PySpike package [45].

To investigate the effect of the electrical stimulation on the neuronal activity, we computed the post-stimulus time histogram—PSTH [32], which represents the average stimulus response of each site. The PSTH was calculated by considering a time window of 400 ms following each stimulus of the train in the recorded signal. We first divided each time window into 4 ms bins and then counted the number of spikes that occurred in each time bin. The probability of the response was then obtained by dividing the spike count per time bin by the number of trial times the bin size. With this definition, all channels with PSTH area below one were removed from the statistical analysis as in [6]. We then computed the percentage of variation of the PSTH area (*PA*) as follows:

(1) $$\Delta PA\;\left[\%\right]=\frac{100*\left(PA_{CCh}-PA_{BAS}\right)}{PA_{BAS}}$$

where *PA_BAS_* is the area of the PSTH computed during the electrical stimulation session in the basal condition (i.e., without any pharmacological treatment), whereas *PA_CCh_* is the PSTH area computed during the electrical stimulation phases in the CCh condition.

We defined as significant a channel that displayed changes in PSTH higher or lower than a specific threshold that was defined based on the ‘stability’ phases. We selected as stability phases the stimulation sessions during the evoked activity without CCh. Specifically, we divided every stimulation session (S1 or S2) into two equal parts of 5 min each (1–5 or 5–10 in the Equation (2)). For each divided session of 5 consecutive minutes, we computed the histogram of the variation in PSTH area (ΔSTIM1, ΔSTIM2) as depicted in Figure 1c. We computed the difference (ΔSTIM1, ΔSTIM2) as follows:

(2) $$\Delta \mathrm{STIM}=PA_{5–10}-PA_{1–5}$$

where *PA*_5–10_ is the area of the PSTH computed during the electrical stimulation session in the last 5 min during basal phase, whereas *PA*_1–5_ is the PSTH area computed in the first 5 min of the baseline during the electrical stimulation phases.

The threshold was defined as the TH20 = mean ± SD of the PSTH area variation (equal to ±20%).

#### 2.4.3. Complexity Indices

The complexity indices aim to quantify the richness of the spatial–temporal patterns displayed by neural networks. Most of them have originally been defined for brain recordings to characterize the brain states in different conditions (anesthesia, stroke, disorder of consciousness, etc.). In the EEG field, Schartner et al. [46] defined a set of indices computed on the spontaneous activities and showed that the brain complexity of healthy subjects undergoing propofol-induced general anesthesia decreased with respect to their awake state. However, those indices well quantified differentiation, while integration cannot, in general, be assessed. Here, instead, we relied on a set of complexity indices designed to measure both the differentiation and integration properties of neural activity. In particular, we computed the ‘neural complexity’ index during spontaneous activity. While there are several definitions of complexity indices computed on the spontaneous activity, much less work has been carried out for evoked activity. The ‘perturbational complexity index’ [20] represents one of the few exceptions. The description of the above-mentioned complexity measures is briefly reported below.

Neural Complexity. The neural complexity (NC) [14] is computed on the spontaneous activity and it measures how much the complementary bipartitions of a network are integrated and segregated. In order to compute NC, at first the instantaneous firing rate (IFR) of each electrode is obtained by binning the spike trains with a bin size of 20 ms. For a given n, the network is subdivided in complementary subsets of n (Xn(t)) and 60-n electrodes (X(t) − Xn(t)), the IFR of the partitions, IFR(Xn(t)) and IFR(X(t) − Xn(t)), is obtained by averaging the IFR of the corresponding electrodes. Finally, the mutual information (MI) is computed between the partitions. The latter procedure is repeated 100 times (*j* = 1, …, 100) to provide a sample of the bipartitions on which MI is computed and finally the average mutual information (<MI>) across samples is retained. This operation is repeated, varying *n* from 5 to 30 (with step 5), and NC is obtained as the sum of the <MI> values.

(3) $$\mathrm{NC}\left(X\right)=\overset{30}{\underset{n=5}{\sum }}<\mathrm{MI}\left(\mathrm{IFR}(X_{j}^{n}\left(t\right)\right);\mathrm{IFR}(X\left(t\right)-X_{j}^{n}\left(t\right)))>$$

The mutual information was estimated by the pyentropy package [47], the firing rates were discretized in 6 levels, and the information bias was corrected with the Panzeri–Treves method (pt in *pyentropy*).

Perturbation Complexity Index. We quantified the perturbation complexity index (PCI) in cortical cell cultures by analyzing the evoked spiking activity. Compared to the human data, cortical cultures yielded a higher signal-to-noise ratio; therefore, we used a smaller number of trials (120) and standard statistics to assess the significant evoked activity. The activity of each channel was binned (bin size = 5 ms) around the stimulation artifact (1000 ms before and 500 ms after the stimulus).

The threshold for significant activation was determined by the bootstrap statistics used in [20] with 500 repetitions, and setting the acceptance value alpha = 0.05. We then extracted a binary matrix of significant sources (SS(x,t)) that represented the spatial (channel x) and temporal (time t after stimulation) activations caused by the electrical perturbation [20]. The channels in the matrix SS(x,t) were sorted from bottom to top on the basis of their total activity during the post-stimulus period (i.e., according to the number of activations). To quantify the minimal amount of redundant information contained in the binary matrix S(x,t), the matrix was compressed using the Lempel–Ziv complexity measure. Finally, PCI is defined as the Lempel–Ziv complexity measure normalized between [0, 1] to make the PCI comparable across conditions (e.g., carbachol treatment). PCI is defined as $\mathrm{PCI}=\frac{C}{\mathrm{Hsrc}\cdot L/\ln\left(L\right)}$, where *C* is the Lempel–Ziv complexity and *L* is a constant (*L* = (number of channels) ∗ (number of time points) = 60 ∗ 100). The term Hsrc is called the source entropy and it is defined as: $\mathrm{Hsrc}=-p_{0}\cdot \ln_{2}\left(p_{0}\right)-p_{1}\cdot \ln_{2}\left(p_{1}\right)$, where *p*_1_ and *p*_0_ represent the fraction of ‘1′ (significant activations) and ‘0′ (non-significant activations).

#### 2.4.4. Statistical Analysis

The data are expressed as mean ± standard error of the mean (SE). Statistical tests were performed to assess the significant difference among the experimental conditions. The normal distribution of data was assessed using Kolmogorov–Smirnov normality test. According to the distribution of data, we performed either parametric (e.g., *t*-test) or non-parametric (e.g., Mann–Whitney) tests, and *p* < 0.05 was considered significant. Statistical analysis was carried out by using OriginPro (OriginLab Corporation, Northampton, MA, USA).

## 3. Results

Each culture of our dataset was first recorded in basal condition (i.e., spontaneous activity) and then underwent electrical stimulation (i.e., evoked response). Analyses were performed both in terms of MUA and LFPs. We then added carbachol (CCh) and repeated the same procedure, in order to investigate both oscillations and spiking during spontaneous activity and in response to stimulation during the drug treatment. As we used two different measures to quantify complexity in our cultures, namely neural complexity (computed during spontaneous, non-stimulated condition), and the perturbational complexity index (computed on the evoked response), we present the results to focus first on the analysis of the spontaneous activity and then on the analysis of the evoked response.

### 3.1. Spontaneous Activity—LFP Analysis

**Figure 2:**
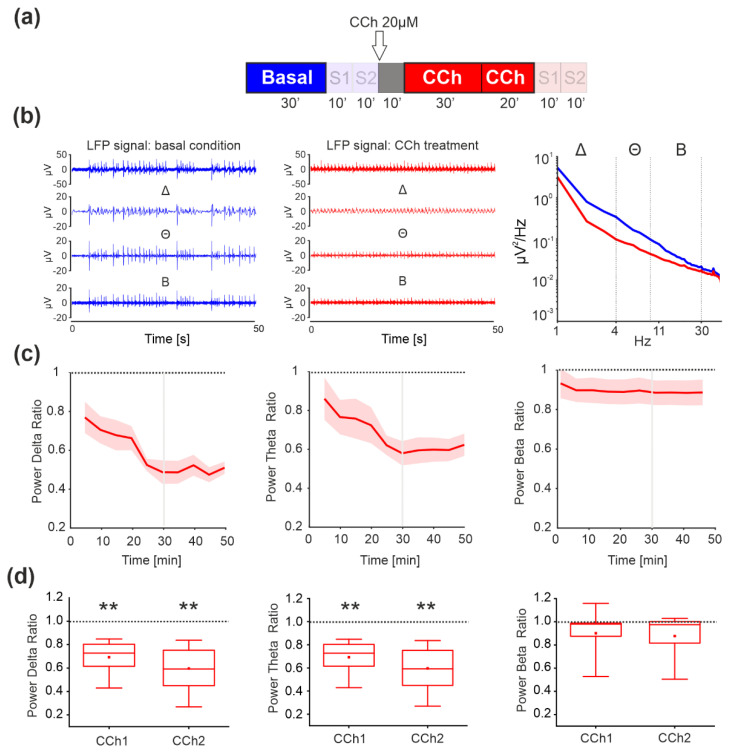
Analysis of local field potentials (LFP) during the basal and CCh phases, to evaluate differences in spontaneous activity. (**a**) Experimental protocol adopted for the experiments. The spontaneous phases (i.e., without electrical stimulation) analyzed here are highlighted. Cortical cultures were recorded for 30′ under basal conditions followed by 1 h of recording after the addition of 20 μM CCh. (**b**) Signal filtered to specific frequency bands involved in sleep for one channel of a representative experiment: local field potential (LFP) signals obtained after band-passing the raw data in the range 1–300 Hz. Different components of the LFP: delta frequency signal (Δ, 1–4 Hz), theta frequency signal (Θ, 5–9 Hz), and beta frequency signal (Β, 10–30 Hz). CCh treatment induced suppression of all low-frequency waves compared to the control condition in one representative experiment (right panel). (**c**) Time course of the power spectral density ratio (CCh over basal) computed in delta, theta, and beta power. (**d**) Box plot comparison of the PSD of each band showing a marked decrease in the delta and theta bands (*n* = 9 MEAs recorded, *p* = 10^−5^). We did not find any difference in the PSD in the beta bands. The bold lines and shaded regions in (**c**) correspond to the mean ± SEM. In each box plot, the small red square indicates the mean, the central red line illustrates the median, and the box limits indicate the 25th and 75th percentiles. Whiskers represent the 5th and the 95th percentiles. Statistical analysis was conducted using the Mann–Whitney comparison test; * *p* < 0.05; ** *p* < 0.01.

We performed experiments using rat cortical cultures plated on MEA. We first evaluated the effect of CCh administration by analyzing the LFPs in absence of electrical stimulation (i.e., Basal, CCh1 and CCh2 phases), according to the protocol depicted in Figure 2a and described in the Methods section.

As reported in Figure 2b (left and center panels), the amplitude of delta, theta, and, to some extent, beta oscillations appeared strongly decreased upon CCh treatment (red profiles). We decided to not include the results on gamma bands since we did not find any difference upon CCh administration in our previous work [27]. Thus, we evaluated how the power spectral density (PSD) changed during the CCh administration. Spontaneous activity was characterized by very slow oscillations in the LFP; indeed, most of the power was concentrated in the delta frequency band (Figure 2b, blue line). Application of CCh to the cultures resulted in a marked decrease in the low-frequency power of the LFP, especially in the delta and theta ranges (Figure 2b, red line). The normalized powers in those frequency bands showed a significant decrease with respect to the basal phase, as indicated by the difference with respect to the dashed line in Figure 2c. In comparing the variation during the first 30 min upon CCh administration (CCh1) and the last 20 min (CCh2), we found a significant decrease in delta and theta power in both phases (Figure 2d, first and second panel *p* = 10^−5^). Beta waves showed a decrease in the power but we did not find any statistically significant difference (Figure 2d, third panel).

### 3.2. Spontaneous Activity—MUA Analysis

**Figure 3:**
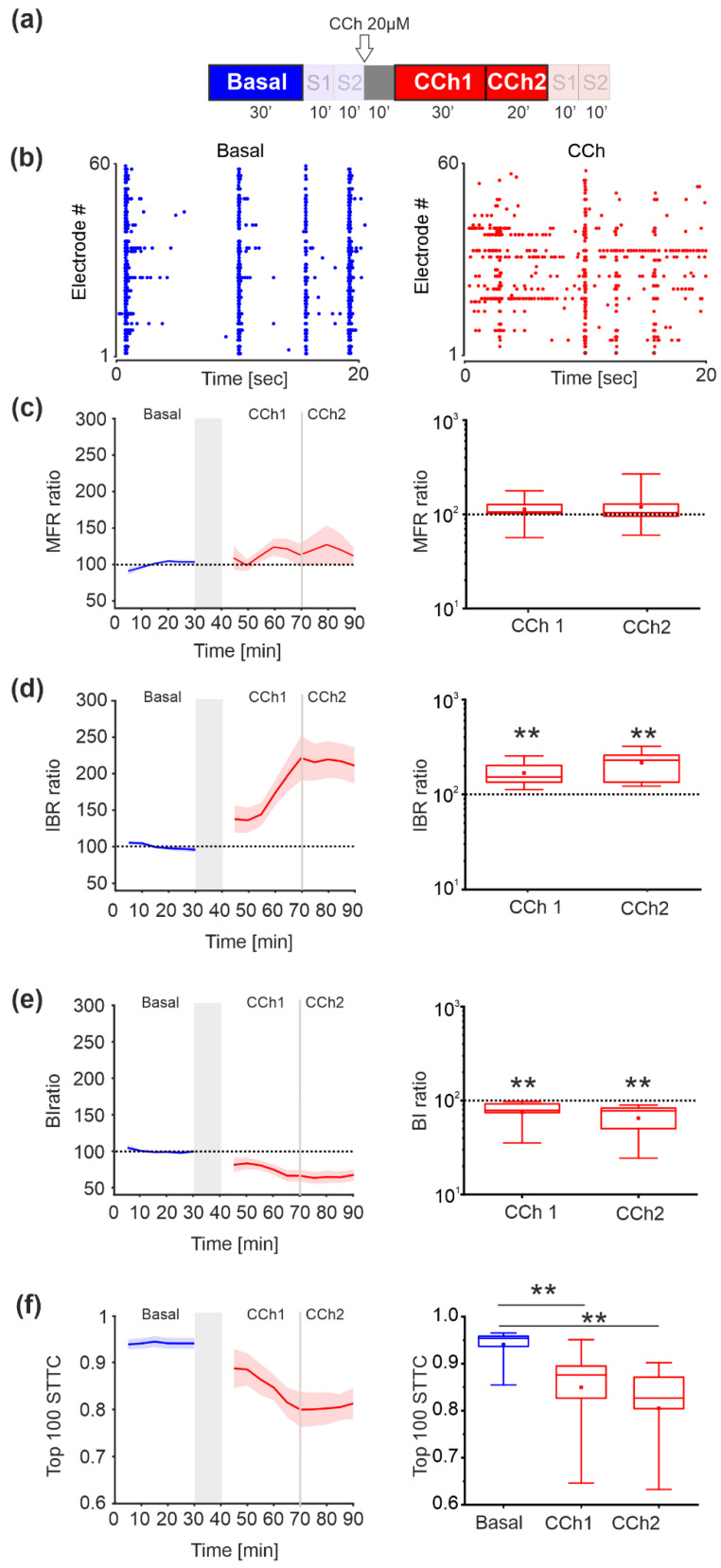
Comparison of the network parameters during basal and CCh administration. (**a**) Experimental protocol adopted in the experiments. The spontaneous phase (i.e., without electrical stimulation), which was analyzed here, is highlighted. Cortical cultures were recorded for 30′ under basal conditions followed by 1 h of recording after the addition of 20 μM CCh. CCh was evaluated at two different time intervals: the first 30′ (CCh1) and the last 20′ (CCh2). (**b**) Raster plots showing 20 s of spontaneous activity (left) and after CCh administration (right) for a representative experiment (each small dot represents a spike, each row an electrode). In the basal condition (blue dots), the activity shows synchronized firing patterns that are completely abolished after the CCh treatment. (**c**) MFR of cultures under basal conditions and during CCh stimulation (*n* = 9). Time-dependent profile (left) and box plot of MFR (right) during CCh treatment. In the group of experiments analyzed here, the MFR of cultures did not change with respect to the basal phase after drug administration (dotted line in the left panel). (**d**) IBR of cultures during basal and CCh stimulation (*n* = 9). Time-dependent profile (left) and box plot of IBR (right) during CCh treatment. In the analyzed group of experiments, the IBR of the cultures significantly increased (*p* = 10^−5^) after drug administration compared to the basal phase (dotted line in the figure). (**e**) BI of cultures under basal conditions and during CCh stimulation (*n* = 9). Time-dependent profile (left) and box plot of BI (right) during CCh treatment (red boxes). In the analyzed group of experiments, the BI of cultures upon drug administration significantly changed (*p* = 10^−5^) with respect to the basal phase (dotted line in the figure). (**f**) STTC values of cultures under basal conditions and during CCh stimulation (*n* = 9). Time-dependent profile (left) and box plot of the STTC values (right) during the basal phase (blue box) and during CCh treatment (red boxes) at two different time intervals: the first 30′ (CCh1) and the last 20′ (CCh2). In the analyzed group of experiments, the STTCs of the cultures increased significantly after drug administration compared to the basal phase (*p* = 0.00276 between basal and CCh1, *p* = 10^−4^ between basal and CCh2). The bold lines and shaded regions (**c**–**f**) correspond to the mean ± SEM. The gray shaded area denotes a 10 min pause in the recording after CCh addition to prevent experimental bias due to drug release into the medium. For each box plot (**c**–**f**), the small square indicates the mean, the central line illustrates the median, and the box limits indicate the 25th and 75th percentiles. Whiskers represent the 5th and the 95th percentiles. Statistical analysis was conducted using the Mann–Whitney comparison test; * *p* < 0.05; ** *p* < 0.01.

According to the protocol depicted in Figure 3a, we then evaluated the effect of CCh administration by analyzing the MUA in the absence of electrical stimulation (i.e., basal, CCh1, and CCh2 phases). In the baseline phase, the activity was characterized by synchronized multi-unit burst activity, as shown in the raster plot of one representative experiment (Figure 3b, left panel). After the administration of CCh, these activity patterns were strongly altered with a fragmentation of burst structures as depicted in Figure 3b, right panel. 

No significant differences in the level of activity expressed by MFR (mean firing rate, spikes/s) were observed during the application of the experimental protocol (Figure 3c). However, CCh application resulted in an increase (*p* = 10^−5^) in the number of isolated spikes (i.e., higher IBR, percentage of spikes outside the bursts, Figure 3d) and a decrease (*p* = 10^−5^) in the burstiness index (BI, Figure 3e) with respect to the basal phase, indicating a loss of bursting activity followed by a loss of synchronicity.

We evaluated the network activity correlation using the STTC method. As shown in Figure 3f, during the basal phase (blue box) the STTC was high since the activity was strongly correlated. After administration of CCh, there was a progressive and significant decrease in the STTC values, indicating desynchronization of the activity (*p* = 0.00276 between basal and CCh1, *p* = 10^−4^ between basal and CCh2). Indeed, the obtained results are consistent with those previously obtained [27] in which electrical stimulation was not part of the protocol.

Moreover, we validated the result also with respect to the SPIKE synchronization measure [44] and we found that spike synchrony was significantly higher in the basal condition (*p* = 0.001, two-way ANOVA with factor drug treatment, synchrony (basal) = 0.271 ± 0.027, synchrony (CCh) = 0.227 ± 0.026, Figure S1).

### 3.3. Complexity in Spontaneous Activity

**Figure 4:**
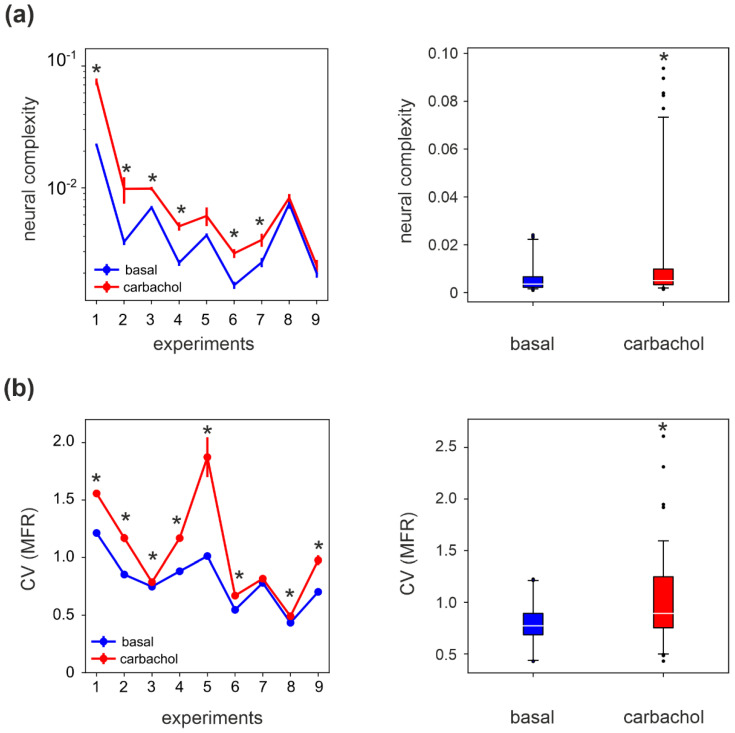
Complexity measures of spontaneous activity. (**a**) Left panel: The neural complexity (NC) during CCh is significantly higher than in the basal condition, in 6 out of the 9 experiments (* *p* < 0.05, *t*-test). Mean and SEM are estimated in sessions of 5 min. Right panel: NC is significantly higher during CCh at the population level (*p* = 0.013, two-way ANOVA with factor drug treatment). (**b**) Left panel: The coefficient of variation (CV) of the channel mean firing rates is significantly higher in CCh with respect to the basal condition in all experiments (* *p* < 0.01, *t*-test). Right panel: CV(MFR) is significantly higher during CCh at the population level (*p* = 10^−4^, two-way ANOVA with factor drug treatment). In the boxplots, the white line corresponds to the mean of the distribution, the lower/upper sides of the box to the first/third quartiles, and the lower/upper whiskers to the 5th/95th percentiles. Black dots in the right panels are outliers, falling below the 5th percentile or above the 95th percentile.

We computed the neural complexity (NC) of the MUA activity. The data were split into segments of 5 min and the NC was computed on each block. We found that NC increased in all experiments, though the increase was only significant in six out of nine experiments (Figure 4a, left panel). By performing a group analysis, we obtained that NC was statistically higher in CCh than in the basal condition (~130% increase Figure 4a, right panel).

Interestingly, the coefficient of variation of the mean firing rate (CV(MFR), Figure 4b, left panel) computed across the MFR of the electrodes also displayed a significant increase in most experiments (eight out of nine). The group analysis indicated that also in the case of CV(MFR), a significant statistical difference was obtained between CCh-modulated and basal activity (Figure 4b, right panel). Indeed, we have previously shown that CCh destabilizes the network’s activity, resulting in a desynchronization of the spiking activity and with an increased variability in the MFR among the electrodes [27]. Coherently, the significant increase in CV(MFR) correlates with the increases in NC in the presence of CCh.

### 3.4. Evoked Activity—LFP Analysis

**Figure 5:**
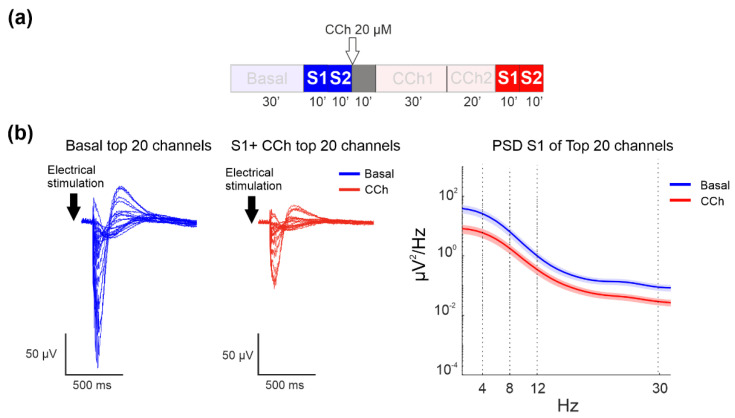
LFP analysis of the evoked response. (**a**) Experimental protocol adopted for the experiments. The electrical stimulation phases analyzed here are highlighted. Cortical cultures were stimulated for 10 min at two different sites under basal conditions and after CCh treatment. (**b**) Left panel: Mean evoked responses of the top 20 channels in one representative experiment during basal conditions and after CCh administration. Right panel: Comparison of the mean PSD of the two different experimental phases: the bold lines and shaded regions correspond to the mean ± SEM.

By considering the phases of electrical stimulation (Figure 5a), we found that CCh treatment also caused strong suppression of the power of the LFP-evoked response, as shown in the evoked LFP signal of the top 20 channels of one representative experiment under the basal condition (blue lines) and after CCh administration (Figure 5b, left panel). The top 20 channels were selected on the basis of their firing rates in the spontaneous phase (i.e., the 20 channels with the highest firing rate in basal condition). CCh caused a decrease in the amplitude without any apparent changes in shape, as shown in the power spectral density evaluation computed for all channels (Figure 5b, right panel).

### 3.5. Evoked Activity—MUA Analysis

**Figure 6:**
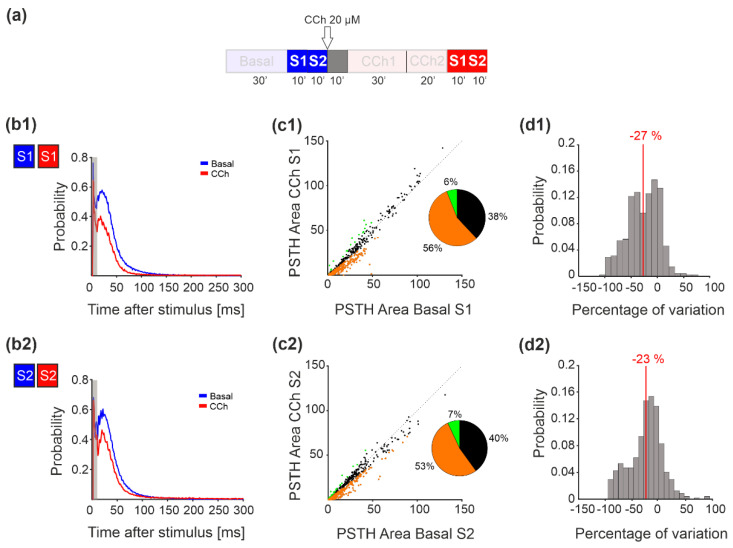
PSTH area analysis. (**a**) Experimental protocol adopted for the experiments. The stimulation phase analyzed here is highlighted. Cortical cultures were stimulated for 10 min at two different sites under basal conditions and after CCh treatment. (**b**) Network PSTH measured in one representative experiment during both stimulation sessions under basal conditions (blue lines) and after CCh treatment (dark red lines). (**c**) The PSTH area of all the experiments for both stimulation sessions showed a higher percentage of channels that decreased relative to the basal level. The variation in each channel was considered significant if it was greater than 20% of the threshold computed by measuring the variation during the stability phases. Orange dots indicate channels that decreased with respect to the basal conditions, green dots indicate channels that increased, and black channels are the ones that did not change. (**d**) The histogram of the percentage of variation for both stimulation sessions showed a mean value centered at −27 for stimulus 1 and at −23 for stimulus 2.

Next, we analyzed the MUA changes due to the effect of electrical stimulation by comparing the PSTH area at baseline with that observed after CCh administration (Figure 6a). Figure 6b depicts the network PSTH observed in one representative experiment during the basal (blue line) and CCh (dark red line) phases for both stimulation sites (S1 in Figure 6b1 and S2 in Figure 6b2). More than 50% of the channels of all the recorded MEAs showed a decrease in the PSTH area compared to the basal condition (Figure 6(c1,c2)). Quantification of PSTH change was carried out according to the threshold TH_20_, as reported in the Methods. We did not find any difference in the PSTH area between the two stimulation sessions, indicating that this reduction did not depend on the order of stimulation. The histogram of the percentage of variation of the PSTH area for each active electrode showed a negative median value for both stimulation sites (Figure 6(d1,d2)).

### 3.6. Complexity in Evoked Activity

**Figure 7:**
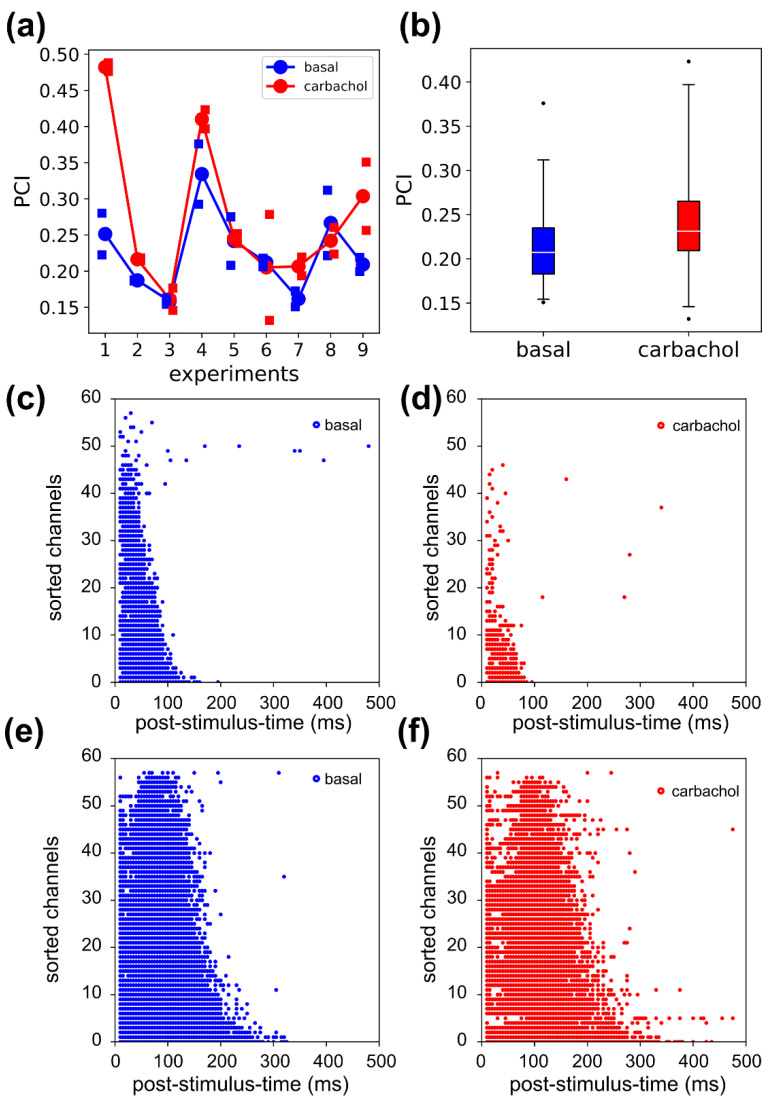
Complexity measure of evoked activity. (**a**) The curves connect the mean PCI obtained by averaging across the PCI values of the two stimuli for each experiment. The squares are relative to the PCI values of the single stimuli. (**b**) The perturbational complexity index (PCI) increases significantly in carbachol by 22% (*p* = 0.023, two-way ANOVA with factor drug treatment). (**c**,**d**) The binary matrix of significant sources relative to exp1, in basal (**c**) and CCh (**d**) conditions. The CCh administration caused a decrease in source entropy (0.55 basal, 0.21 CCh). (**e**,**f**) In exp9, the binary matrices of significant sources have comparable source entropies across conditions (0.92 basal, 0.93 CCh). The dots in the panels (**c**–**f**) correspond to the significant spatial-temporal activations.

PCI was computed for the both the S1 and S2 stimuli (Figure 7a) on the spiking activity. At the population level, we found that PCI increased after CCh administration (*p* = 0.023, percentage increase = 22%, Figure 7b). We noted that experiment 1 was quite peculiar with respect to the other experiments, as the large PCI increase was paired with a corresponding large decrease in the source entropy. The significant activations (Figure 7c,d) under CCh were reduced and visibly did not display more complex spatial–temporal patterns with respect to the basal condition. As a comparison, consider experiment 9 (Figure 7e,f) in which the source entropy stayed almost the same (basal 0.92, CCh 0.93) and PCI increased (basal 0.22, CCh 0.35). In the latter case, the significant activations were slightly more sparse in space and time, which could explain the PCI increase. Based on the considerations, we hypothesized that experiment 1 was an outlier, and once this was excluded, the PCI dropped to 11% and was no longer different from the basal condition (*p* = 0.067).

In order to verify the result, without depending on the type of signal (i.e., MUA) used for the computation of PCI, we also calculated it for the band-pass filtered 1–300 Hz data. We kept all parameters of the bootstraps statistics the same and we found no significant changes between the PCI values (*p* = 0.68 two-way ANOVA test with factor drug treatment, PCI_LFP_ (basal) = 0.207 ± 0.013, PCI_LFP_ (CCh) = 0.214 ± 0.011, Figure S2).

## 4. Discussion

Brain complexity has been quantified in human studies, both during resting state [46] and under perturbation [20]. It has been also explored in cortical brain slices, at the microscale level, where the anatomical circuitry of the tissue of origin is preserved [25].

Here, for the first time, we investigate complexity in a simplified experimental model in vitro, in which neurons spontaneously establish connections and create random-like networks without reproducing a specific anatomical structure and its related connectivity. Clearly, one cannot compare the values of complexity found in slices, let alone in cell cultures, with those validated in humans, in part because they are obtained with different techniques at different scales [48]. Instead, our goal here was to understand whether complexity computed in cortical cultures, both during spontaneous and evoked activity, could be manipulated in ways that mimic the effects of activating systems in more structured brains.

In our study, we administered carbachol (CCh), which was able to modulate sleep-like properties in vitro [25,27,29]. During sleep, in both humans and rodents, network activity is synchronized across brain areas, while during wakefulness it is desynchronized [49,50,51,52]. Consistent with this behavior, the administration of CCh caused desynchronization of the spiking activity and suppression of the low-frequency oscillations, which are dominant during sleep [27,53,54,55,56]. In this regard, the spectral exponent recently introduced in human EEG recordings [57] shows that the fit of the decay of the broadband EEG spectrum can distinguish between the conscious and the unconscious state (the slope becomes steeper in the latter case). Interestingly, in Figure 2b, the decay of the spectrum is flatter in the CCh condition than in the basal one, reflecting a departure from the sleep-like state of the cultures.

At first, we analyzed the spontaneous regime in control and CCh conditions. We found that neural complexity (NC) [14] significantly increased upon treatment with CCh. The distribution of firing rates in the network widened under CCh (i.e., higher coefficient of variation), suggesting that the increase in NC could be determined by the higher ‘noise’ under conditions of increased excitability of individual neurons. To corroborate this hypothesis, the overall population activity was functionally less aggregated under CCh, as confirmed by the weakened correlation strengths. Indeed, in a system of non-interacting elements (i.e., where strong lateral connections are missing) measures of entropy can show very high values, although the system under analysis lacks a complex causal structure [15].

Such dissociation between the complexity of observable dynamics and the actual complexity of causal interaction was unequivocally confirmed by analyzing the population responses to direct perturbations. We delivered electrical stimulation to our neuronal cultures using a low-frequency stimulation regime (i.e., 0.2 Hz) to avoid inducing long-term plasticity [34]. Indeed, no changes in the evoked response, in the level of synchronization, or in the firing rate activity were observed, as also reported in the literature after several sessions of stimulation [6,33,35]. Conversely, stimulation frequency >1 Hz can induce different firing regimes [58], long-term potentiation, and depression [6,59].

Here, similarly to slice, rodent, and human experiments, the initial response was smaller under the effect of activating neuromodulation; however, cortical cultures failed to show the typical resurgence of complex recurrent interactions. The first effect, evident from smaller PSTH, LFP, and spectral responses (Figure 5 and Figure 6) reflects changes in responsiveness at the level of single neurons, whereby depolarization may dampen the initial response due to decreased burstiness and a weaker driving force for depolarizing currents. The second effect, instead, reflects network properties and reveals key differences between dissociated cortical cultures and in vitro/in vivo models with preserved architectures.

Indeed, while in cortical slices and intact brains awakening or activating neuromodulation invariably results in the emergence of a sequence of waves of activity with a complex spatial-temporal distribution, in the case of cell cultures, the shape of the MUA-related and LFP responses to the stimulation was quite similar, even after the application of CCh. In fact, we noticed a slight decrease in the response to perturbation in terms of oscillations (i.e., LFP analysis). Accordingly, computing PCI in the cultures revealed only a moderate increase in PCI (~20%) under CCh, considerably lower than the changes observed in slices (~80%, [25]) and in the human brain (~100%, according to [21], where comparison between NREM sleep to wakefulnessis reported). The present disconnection between the complexity of spontaneous activity (NC) and the complexity of causal interactions (PCI) in neuronal cultures bears a general relevance and deserves further investigation. For example, it suggests that the enabling factor represented by activating neuromodulation may not be sufficient if the network lacks the appropriate architecture (i.e., is segregated or random, instead of grid-like) [16,17]. It also prompts a reflection on how different measures of complexity (i.e., spontaneous vs. perturbational) capture different aspects of the system under study, warranting caution when making inferences [15].

Along these lines, an interesting addition to our experimental design could be to use a cocktail of drugs [25,54], instead of CCh alone, to better resemble the landscape of frequency oscillations observed in the awake human brain. An even more intriguing perspective is linked to the possibility to artificially ‘drive’ the connectivity of the cells within the cultured network. Indeed, cell cultures are not necessarily random networks [9,60], and, thanks to their versatility, one can manipulate neuronal growth to obtain networks with different topologies [61,62,63], even with a 3D architecture [64,65,66]. This peculiarity allows us to design networks [67] characterized by recurrent connections and feedback loops more akin to those found in the thalamo-cortical circuit. Finally, brain organoids represent the most recent and promising experimental preparation to study brain complexity across states. They feature spontaneous neural oscillations typical of in vivo systems, that can be modulated with different type of drugs. During development, brain organoids mimic the corticogenesis process, allowing for the formation of 3D networks resembling those of the real brain. Importantly, given the progress in the field, some authors [68] also raised ethical questions about the possibility that brain organoids could one day experience a conscious state. Although, even if there are still known reproducibility issues across different studies [69] and recording electrophysiology signals from 3D networks still faces important issues and needs to be ameliorated [70], brain organoids will be a fundamental experimental preparation for many studies in the neuroscience field, including the investigation of complexity. 

In the above perspective, recordings and perturbations in cell cultures may provide interesting insights on the minimum requirements (in terms of micro and meso-scale connectivity and neuromodulatory milieu) that are jointly needed for the emergence of the kind of complexity that is found to be relevant in humans across physiological and pathological brain state transitions.

## Data Availability

The data presented in this study are available on request from the corresponding author.

## Supplementary Materials

The following are available online at https://www.mdpi.com/article/10.3390/brainsci11111453/s1, Figure S1: Spike synchronization, Figure S2: PCI computed on the LFP signal.
