# Supplementary material for: Spontaneous and Perturbational Complexity in Cortical Cultures
Source: Brain Sci. 2021 Nov 1;11(11):1453. doi: 10.3390/brainsci11111453 (PMC8615728; doi:10.3390/brainsci11111453)
Supplement: Supplementary file 1 [file brainsci-11-01453-s001.zip › brainsci-1421525-supplementary.pdf]

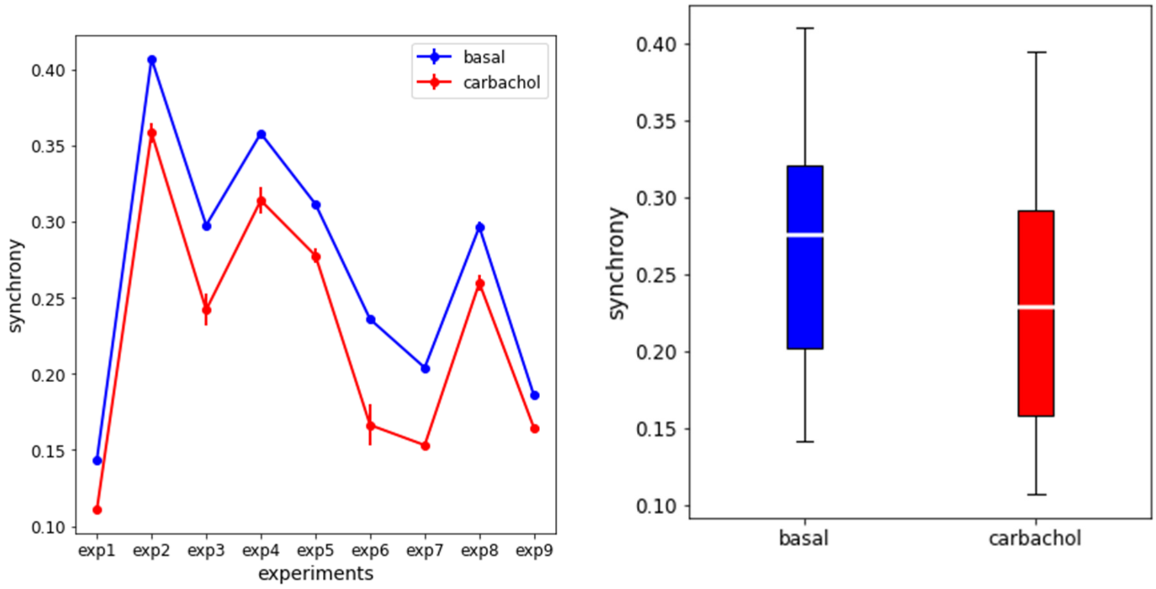

(a) (b)

Figure S1. Spike synchronization. (**a**) The spike-synchronization is systematically higher in the basal condition with respect to carbachol. (**b**) At the population level the spike-synchrony is significantly higher in carbachol (Anova two-way with factor drug-treatment p=0.001).

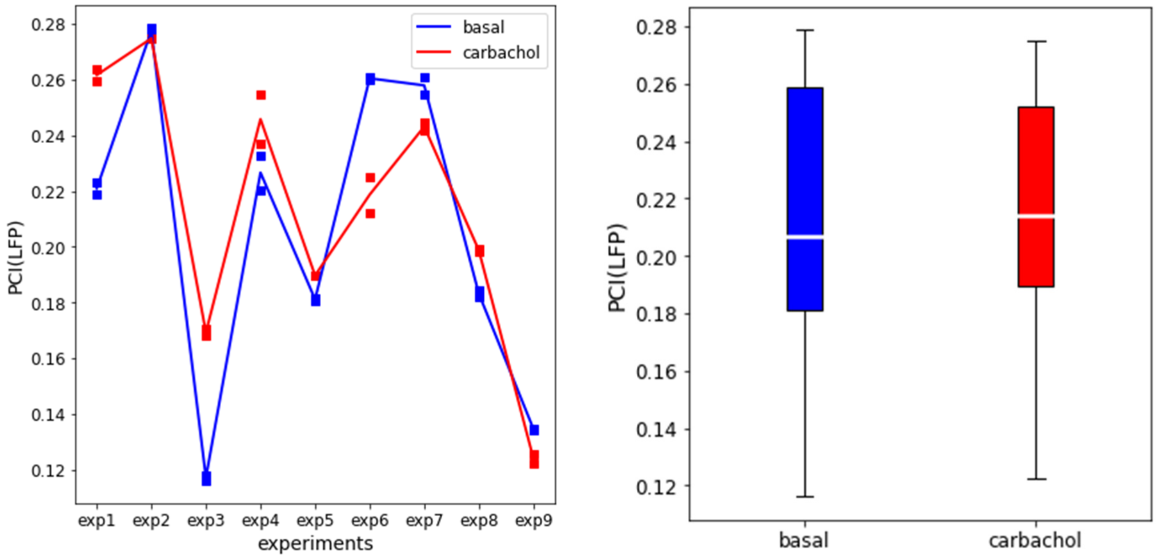

(a) (b)

Figure S2. PCI computed on the LFP signal. (**a**) PCI is computed for the S1,S2 stimuli (square symbols) of all experiments. The continuous lines pass through the averaged PCI for the stimuli S1 and S2. (**b**) The slight increase of PCI is not significant at the population level (p=0.68 two way Anova test with factor drug treatment).
